# Measurement and projection of the burden of disease attributable to population aging in 188 countries, 1990-2050: A population-based study

**DOI:** 10.7189/jogh.12.04093

**Published:** 2022-10-30

**Authors:** Jun-Yan Xi, Xiao Lin, Yuan-Tao Hao

**Affiliations:** 1 Department of Medical Statistics, School of Public Health, Sun Yat-sen University, Guangzhou, China; 2 Peking University Center for Public Health and Epidemic Preparedness & Response, Beijing, China; 3 Sun Yat-sen Global Health Institute, Sun Yat-sen University, Guangzhou, China; 4 Center for Health Information Research, Sun Yat-sen University, Guangzhou, China

## Abstract

**Background:**

Quantitative attribution of the burden of disease due to population aging is an important part of setting meaningful global health priorities. This study comprehensively examines the burden of disease attributable to population aging in 188 countries from 1990 to 2019, incorporates a comprehensive range of diseases, and projects the burden of disease due to population aging till 2050.

**Methods:**

We extracted data from 1990 to 2019 for 188 countries from the Global Burden of Disease Study 2019. We decomposed the change in disease burden into the contribution of the age structure of the population, population size, and age-specific disability-adjusted life years (DALYs) rates due to all other reasons. We used the Bayesian age-period-cohort model to evaluate the effects of age on temporal trends, and then to predict the possible disease burden in 2050.

**Results:**

At the global level, the change in total DALYs associated with age structure, population size, and all other reasons is 27.4%, 16.8%, and 89.4% (absolute level of DALYs attributable to age structure: -15.20 million, 9.32 million, and -49.58 million) of the absolute level of DALYs gap between 2019 and 1990. The absolute level of DALYs changes attributable to age structure for communicable, maternal, neonatal, and nutritional diseases were negative in all income groups from 1990 to 2019. For non-communicable diseases, the contribution was positive except in the low-income group. For injuries, the contribution was positive in lower-middle-income groups and low-income groups. By 2050, DALY rates decreased in all income groups, if compared to 2019. However, a total of 132 countries may see a gradual increase of all-cause DALYs attributable to population aging.

**Conclusions:**

The direction and intensity of the effects of population aging on the burden of disease vary by region and disease, with huge implications for global health in the future.

The world is facing the complex challenge of population aging [1]. Life expectancy at birth has risen markedly worldwide since 1990, although with different temporal patterns of this trend [2]. The new demography of low fertility and low mortality produces population aging. Between 2020 and 2050, the worldwide population of people aged 60 and over will double to 2.1 billion, and the number of people aged 80 and over will triple to 426 million [3]. The increase in life expectancy is to be celebrated as an achievement of civilization and brought more potential opportunities for society. Older people who live longer may engage in new activities of personal interest and contribute to their families or society [4]. However, these opportunities and contributions are heavily dependent on the health of the extra years of life. Older people are the most vulnerable groups to disease and disability [5,6]. The increasing number of older people will exacerbate the global burden of disease because the elderly may have a higher demand for health services than younger people [7]. The burden of disease attributable to population aging will therefore have a fundamental effect on the sustainability of modern society.

Quantitative attribution of the burden of disease due to population aging is an important part of setting meaningful global health priorities [8]. Since 1990, the Global Burden of Disease Study (GBD) has provided comprehensive estimates of disability-adjusted life years (DALYs) to address the changes in the burden of disease. GBD 2017 decomposed the changes of DALYs related to 84 risk factors from 2007 to 2017, and the results showed that population aging resulted in an increase in DALYs related to all causes, non-communicable diseases, and injuries, and a decrease in DALYs related to communicable, maternal, neonatal, and nutritional diseases (CMNNDs) [9]. Some studies have applied fixed historical rates of incidence or mortality to the population projections to examine the changes occurring in the demographic composition that leads to an increased burden of disease for some diseases [10,11]. However, previous studies have included only a limited number of countries, and diseases and methodological differences may make the results incomparable, making it less sufficient to fully reflect the regional differences and disease differences of population aging. Another challenge is the lack of global projections of DALYs attributable to population aging. Full consideration of possible changes in DALYs as a result of population aging would provide useful information for governments worldwide to move with urgency to introduce health-related policies.

To sum up, this study focuses on the following objectives. First, we analysed the changes in DALY by estimating the age, period, and cohort effects to segregate and examine the effect of increasing age on the burden of disease. Second, we decomposed the DALY changes for different categories of diseases among 188 countries from 1990 to 2019 into the effects of the age structure of the population (a proxy for population aging), population size, and age-specific DALYs rates due to all other reasons. Third, we forecast global DALYs attributable to population aging till 2050.

## METHODS

### Data sources

We obtained standard epidemiological measures from the GBD 2019 for the three cause groups (Level 1) and 22 cause subgroups (Level 2) under the GBD cause hierarchy by country and year, including demographics, years of life lost (YLLs), years lived with disability (YLDs), and DALYs. GBD 2019 is a multinational collaborative study that estimates the burden of diseases in countries around the world and produces summary measures of health. The study updates the data sets annually, thus consistent comparisons could be made between sex, age, and across locations from 1990 to 2019. The data sources and methods are described elsewhere [12]. All data sets are available via the GBD 2019 website [13].

Population projections for 2020 to 2050 were obtained from the medium variant project of the World Population Prospects 2019 published by the United Nations (UN) Population Division. This data set provides the median and 95% prediction intervals with quinquennial population by country, sex, and five-year age groups (0 to 4, 5 to 9, 10 to 14, ..., 95 to 99, 100+), and it is estimated using the Bayesian hierarchical models based on the total fertility rates and life expectancy at birth. Detailed information concerning the UN methods can be extracted elsewhere [14]. The data set is also available online [15].

In addition, we grouped selected countries based on the World Bank's classification, including high-income countries, upper-middle-income countries, lower-middle-income countries, and low-income countries [16]. This study complies with the Guidelines for Accurate and Transparent Health Estimates Reporting (GATHER) statement [17].

#### Attribution decomposition method of population aging

We refer to the approach developed by Cheng et al. [18] to ascribe differences in DALYs to the contribution of three factors: age structure of the population, population size, and age-specific DALYs rates due to all other reasons. This method overcomes the limitation of two established and widely used methods [19,20]. That is, the inconsistent decomposition results when the reference population is altered due to the asymmetric allocations of interactions between relevant factors. Model details are presented in the **Online Supplementary Document**.

#### Bayesian age-period-cohort regression model

The Bayesian age-period-cohort (APC) regression model was conducted to evaluate the effects of age on temporal trends, and then to predict the possible disease burden in 2050. The collinearity among the three factors causes the model unidentifiable and a parameterization method by setting constraints on the parameters to overcome dependence suggested by Holford [21]. We constrained cohort effects to be 0 on average with 0 slope, and the inclusion of the drift with the period effect makes the age effect interpretable as the age-specific rates in the reference period adjusted by the cohort effect. The model was fitted using B-spline and the relevant sub-models (the linear, nonlinear cohort, and period effects) were provided to test the goodness of model fit by comparing the residual deviance. To efficiently forecast future rates and counts within a fully Bayesian inference setting, we chose the second-order random walk priors (RW2) for age, period, and cohort effect [22]. These priors penalize deviations from a linear trend with better predictions. The hyper prior for the precision (inverse variance) in the random walk priors is a Gamma distribution with parameters a and b, and weak hyperparameters (*a* = 1, *b* = 0.00005) were set to smooth the estimation [23]. Model details are presented in the **Online Supplementary Document**.

#### Uncertainty analysis

We computed the 95% uncertainty intervals (UIs) by propagating the uncertainty from the GBD 2019 estimates. Specifically, we calculated from 1000 draw-level estimates using the Monte Carlo method, assuming that the input parameters complied with the triangular distribution [24]. 95% UIs were estimated using the 2.5th and 97.5th percentiles of the draws.

## RESULTS

### Proportion of the population aged over 65 in 1990 and 2019

**Figure 1:**
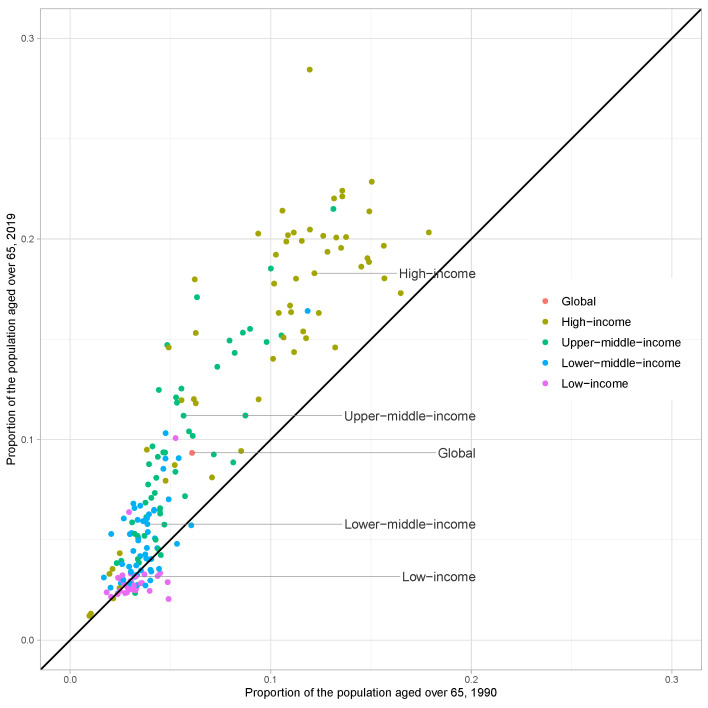
Proportion of the population aged over 65 in 1990 and 2019.

Considering the age structure of the population, as measured by the proportion of the population aged 65 and over, only the low-income group has seen a decrease between 1990 and 2019. For individual countries, 19 low-income countries, 14 lower-middle-income countries, two upper-middle-income countries (Equatorial Guinea and Gabon), and one high-income country (Oman) have slowed population aging. As of 2019, 88 out of 188 countries have been defined as aging societies, with the proportion of the population aged 65 or above exceeding 7% (including the global group, upper-middle-income group, and high-income group) (**Figure 1**).

#### Age, period, and cohort effects of trends in DALY rates from 1990 to 2019

**Figure 2:**
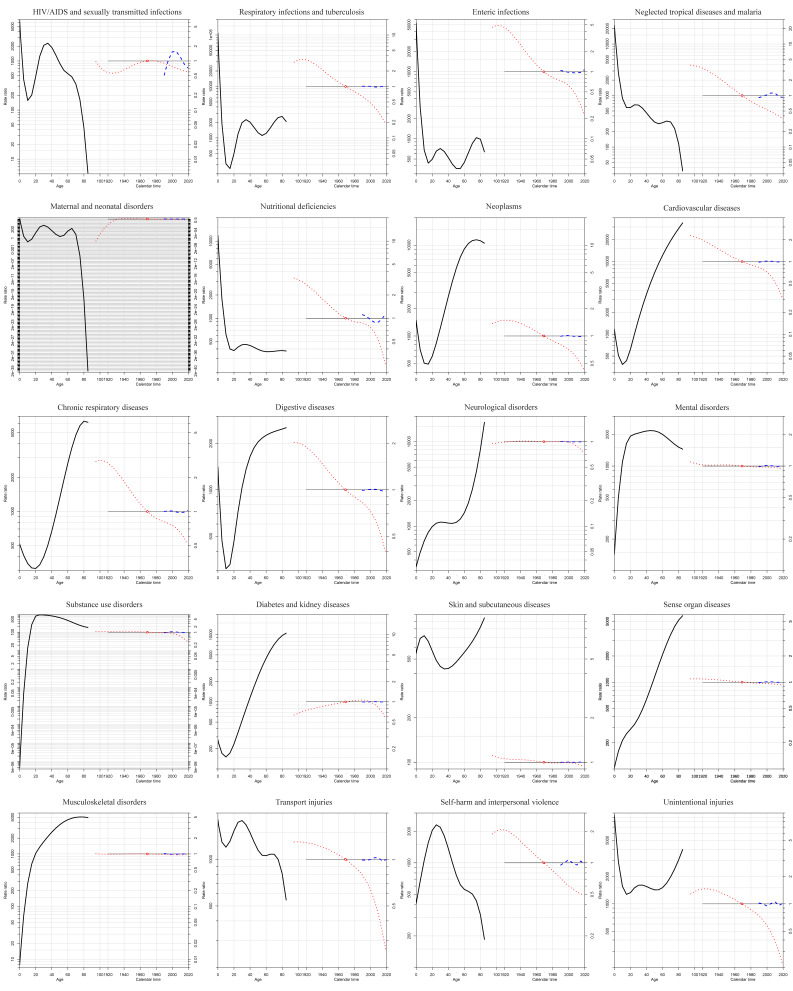
Age, period, and cohort effects of trends in DALY rates from 1990 to 2019. The solid black line, dotted blue line and dotted red line represent the age effect, period effect and cohort effect respectively.

**Figure 2** revealed the effects of age, period, and birth cohort by disease clusters globally. For some CMNNDs, the DALYs rate ratio fluctuates slightly with age, but there are exceptions. The DALYs rate ratio of HIV/AIDS and sexually transmitted infections, for example, peak at age 35 and then declines. The DALYs rate ratio of neglected tropical diseases and malaria continues to decline with age. Meanwhile, different from the period effect of the relatively stable in other diseases, the fluctuations in period effects can be observed in HIV/AIDS and sexually transmitted infections, neglected tropical diseases and malaria, and nutritional deficiencies. In addition, the DALYs rate ratio for almost all non-communicable diseases increases rapidly with age and has a relatively stable period effect. However, the peak DALYs rate ratio reached at the age of 45 years old for mental disorders and 25 years old for substance use disorders, and then decline slowly with age. DALYs rate ratio of transport injuries and self-harm and interpersonal violence decreased with age, but unintentional injuries showed the opposite trend. Except for HIV/AIDS and sexually transmitted infections, where a fluctuating cohort effect was observed, the cohort effect continued to decline or remained relatively stable for almost all diseases.

#### Changes in DALYs attributable to population aging from 1990 to 2019, by disease clusters and income groups

**Figure 3:**
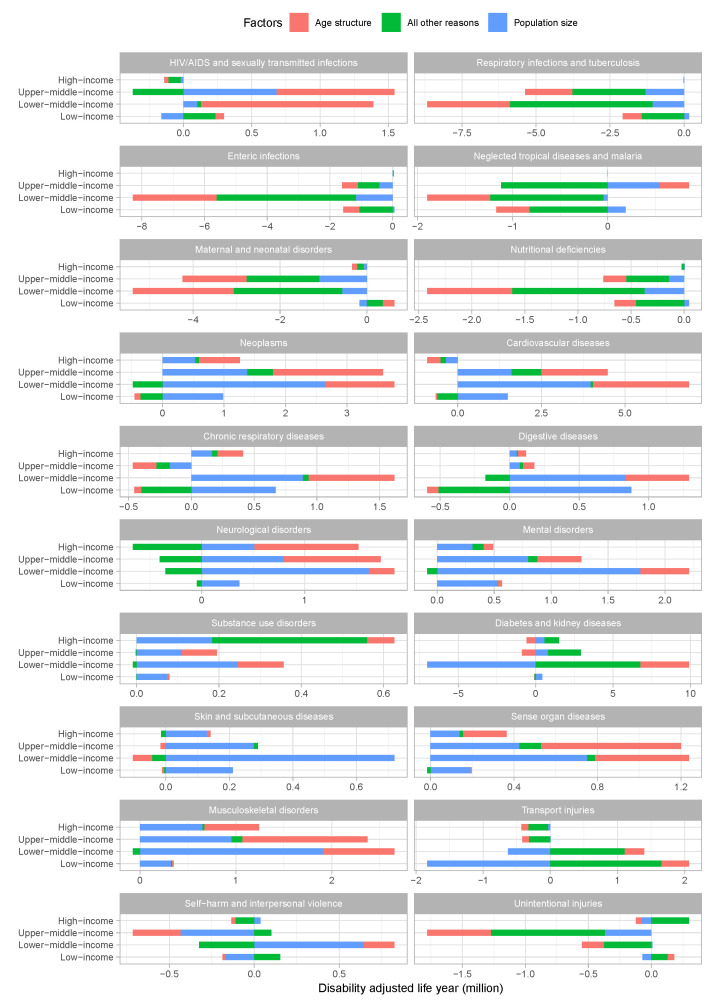
Changes in DALYs attributable to population aging from 1990 to 2019, by disease clusters and income groups.

At the global level, the change in total DALYs associated with age structure is 27.4% (absolute level of DALYs attributable to age structure: -15.20 million) of the absolute level of DALYs gap between 2019 and 1990, -16.8% (9.32 million) of the change associated with population size, and 89.4% (-49.58 million) of the change associated with age-specific DALYs rates due to all other reasons. Absolute level of DALYs changes attributable to age structure for CMNNDs were negative in all income groups from 1990 to 2019 (17.5% for percentage of change, with absolute DALYs being -0.98 million for high-income group; 32.3%, -41.24 million for upper-middle-income group; 34.9%, -116.08 million for lower-middle-income group; and 36.3%, -24.90 million for low-income group). However, for HIV/AIDS and sexually transmitted infections, except for the high-income group (23.0% for percentage of change, -0.32 million for absolute DALYs), the absolute level of DALYs changes attributable to the age structure of other income groups were all positive (73.0% for percentage of change, with absolute DALYs being 8.58 million for upper-middle-income group; 90.7%, 12.61 million for lower-middle-income group; and 46.0%, 0.62 million for low-income group). In addition, the positive absolute level of DALYs changes attributable to age structure was also found for enteric infections in the high-income group (35.6% for percentage of change, 0.12 million for absolute DALYs), neglected tropical diseases and malaria in the upper-middle-income group (-114.6%, 3.07 million), and maternal and neonatal disorders in the low-income group (58.4%, 2.69 million). Absolute level of DALYs changes attributable to age structure were positive for non-communicable diseases except in low-income group (45.9% for percentage of change, with absolute DALYs being 25.54 million for high-income group; 46.8%, 70.60 million for upper-middle-income group; 33.4%, 83.23 million for lower-middle-income group; and -53.4%, -23.49 million for low-income group). However, some contrary results were shown in subgroup analyses, such as positive absolute level of DALYs changes attributable to age structure for mental disorders, substance use disorders, and musculoskeletal disorders in the low-income group (6.3% for percentage of change, 0.36 million for absolute DALYs; 8.4%, 0.07 million; and 5.0%, 0.18 million), and negative for diabetes and kidney diseases in the high-income group and upper-middle-income group (-65.8%, -6.12 million; -47.1%, -9.58 million). For injuries, absolute level of DALYs changes attributable to age structure were negative in high-income and upper-middle-income groups (15.6%, -0.55 million; and 24.5%, -6.89 million), and positive in lower-middle- and low-income groups (32.6%, 2.37 million; and 58.9%, 1.79 million). The exception is that the absolute level of DALYs changes attributable to age structure for self-harm and interpersonal violence in the low-income group (50.5%, -0.17 million) and unintentional injuries in the lower-middle-income group (32.3%, -1.75 million) are both negative (**Figure 3**). The changes in DALYs attributable to population aging from 1990 to 2019 with 95% UIs by disease clusters and country are provided in the Supplementary Data 1 file available at https://github.com/admin-xi/JoGH-3147-Supplementary-Data.git.

#### Changes in YLLs and YLDs attributable to population aging from 1990 to 2019, by disease clusters and income groups

**Figure 4:**
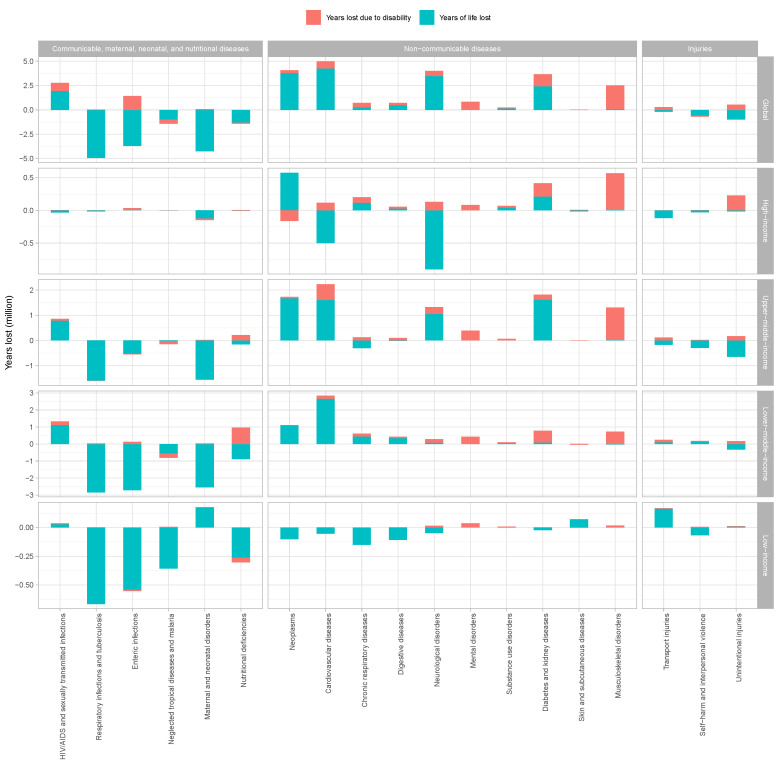
Changes in YLLs and YLDs attributable to population aging from 1990 to 2019, by disease clusters and income groups.

We conducted attributional decomposition of population aging for YLLs and YLDs respectively. Overall, YLLs of -104.55 million (27.6% for age structure contribution of YLLs) and YLDs of 92.83 million (28.7% for age structure contribution of YLDs) constitute the global absolute level of DALYs changes in attributable to age structure. A higher proportion of YLLs is attributable to age structure than YLDs in CMNNDs. In subgroup analysis, both YLLs (accounted for 71.0%) and YLDs (accounted for 29.0%) attributable to the age structure of HIV/AIDS and sexually transmitted infections have a positive contribution (88.3% for percentage of change, 19.61 million for absolute DALYs; 234.7%, 7.99 million), and other income groups similar except high-income group. In contrast to reduced YLLs, Respiratory infections and tuberculosis (15.5%, 0.31 million), enteric infections (495.3%, 14.37 million), and maternal and neonatal disorders (7.4%, 0.83 million) were positive YLDs attributable to age structure. For non-communicable diseases, the proportion of YLLs and YLDs attributable to age structure accounted for 53.7% and 46.3% respectively (46.1%, 100.43 million; 30.6%, 86.52 million). In subgroup analysis, chronic respiratory diseases, mental disorders, and musculoskeletal disorders have a higher proportion of YLDs attributable to age structure than YLLs. For injuries, the absolute level of YLLs (29.2%, -12.64 million) and YLDs (41.3%, 9.12 million) attributable to age structure showed an opposite trend globally. The exception was the negative YLDs attributable to age structure for self-harm and interpersonal violence (-39.0%, -0.90 million). There are remarkable differences between income groups (**Figure 4**). The changes in YLLs and YLDs attributable to population aging from 1990 to 2019 with 95% UIs by disease clusters and country are provided in the Supplementary Data 1 file available at https://github.com/admin-xi/JoGH-3147-Supplementary-Data.git.

#### Projections of global total DALY rates and the attributable aging burden in 2050

**Figure 5:**
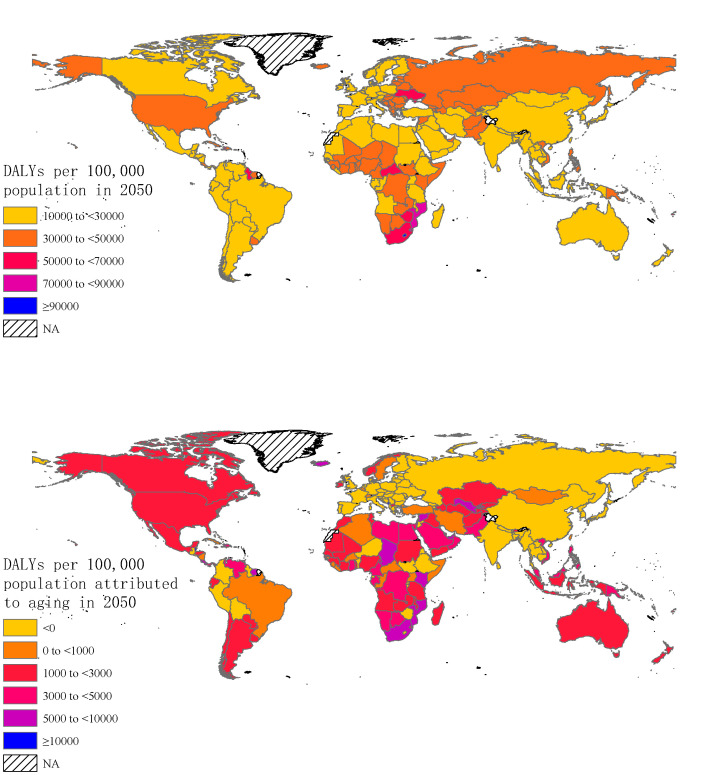
Projections of global total DALY rates and DALY rates attributable to age structure in 2050. NA = not available.

The global probable total DALY rates in 2050 are 29 041.68 person-years (DALYs per 100 000 population), which is lower than 32 801.70 person-years in 2019. Possible DALY rates were 27 430.33 person-years for the high-income group, 26 620.15 person-years for the upper-middle-income group, 30 451.83 person-years for the lower-middle-income group, and 33 851.80 person-years for low-income group, respectively. DALY rates decreased in all income groups compared to 2019 (DALY rates in 2019 were 29 757.90 person-years for the high-income group, 28 425.91 person-years for the upper-middle-income group, 34 732.99 person-years for the lower-middle-income group, 45 653.23 person-years for the low-income group). Total DALY rates may exceed 90 000 person-years in 11 countries, including the United States Virgin Islands, Micronesia (Fed. States of), Antigua and Barbuda, Grenada, Tonga, Saint Vincent and the Grenadines, Seychelles, Kiribati, Lesotho, Guam, and Saint Lucia. The reference point for DALY rates attributable to age structure is 2019. The global rates of DALY attributable to age structure were 1300.28 person-years. By income, they were 267.45 person-years for the high-income group, -120.13 person-years for the upper-middle-income group, 1551.11 person-years for the lower-middle-income group, and 1950.34 person-years for the low-income group, respectively. 65 countries will have negative DALY rates attributable to age structure, ranging from -38 952.67 person-years to -88.82 person-years (**Figure 5**).

**Figure 6:**
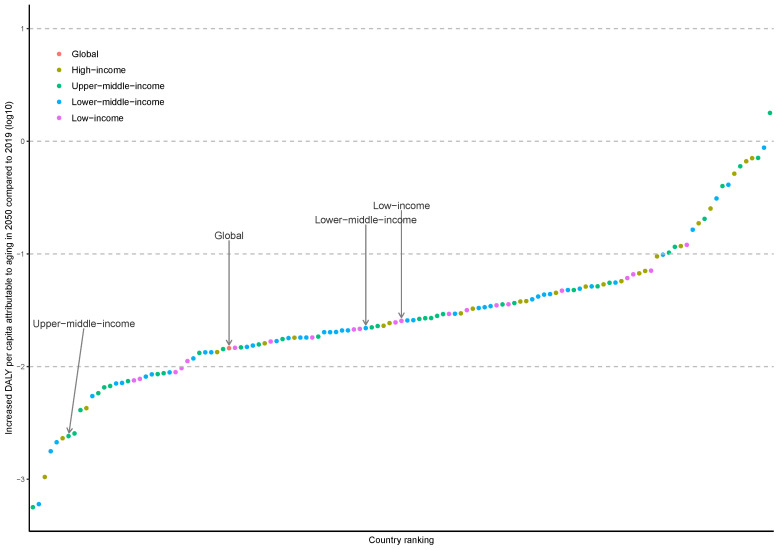
Per capita DALYs needed to be controlled to offset the negative effect of population aging in 2050.

Considering the negative effect of population aging, as measured by the difference of absolute level of DALYs changes attributable to age structure between the two periods is greater than 0 (2019-2050 vs 1990-2019), 132 countries were included. Age structure changes had a negative effect on absolute level of DALYs at global level and in three income groups (1456.41 person-years, 95% UIs = 1388.53-1530.42 person-years for global; 219.21 person-years, 95% UIs = 207.09-232.58 person-years for upper-middle-income group; 2195.81 person-years, 95% UIs = 2038.20-2372.32 person-years for lower-middle-income group; and 2544.06 person-years, 95% UIs = 2390.45-2722.53 person-years for low-income group) (**Figure 6**). The changes in DALYs attributable to population aging from 2019 to 2050 with 95% UIs by disease clusters and country are provided in the Supplementary Data 2 file available at https://github.com/admin-xi/JoGH-3147-Supplementary-Data.git.

## DISCUSSION

The decline in global age-standardized DALY rates over the past 30 years obviously, indicates that global health is improving as expected [12]. Population growth and aging are often seen as two important factors preventing the reduction of disease burden, which may overshadow the great achievements in public health. However, our analysis showed that population size and age structure play different roles for various populations and diseases. We found that relative to age structure and all other reasons, population size was the only factor driving the increase in the absolute level of DALYs from 1990 to 2019. This result is also supported by Angela Y Chang et al. [8]. However, age structure change is still identified as the main driving of the absolute level of DALYs, and its effect is higher than population size. We observed that the absolute level of DALYs attributable to age structure was mainly the decrease in death burden and the increase of disability burden. The potential consequence is a further expansion of life expectancy (LE) and health-adjusted life expectancy (HALE). The gap between LE and HALE was used to measure years lived in poor health, symbolizing extra years of life with a low quality of life [25]. Narrowing this gap to parallel increases in LE and HALE should be one of the most important and urgent agendas for healthy aging [26].

Our analysis found that the reduced burden of premature death from CMNNDs is an achievement of an aging society. This substantial trend has been observed in most countries in the Asian and African regions. It is remarkable, however, that considerable heterogeneity within the African region. Some African countries, such as South Africa and Congo, lead the world in increased attributable premature mortality from CMNNDs. Interpretation of spatial patterns needs to take into account the fact that some factors have a strong relationship with population characteristics. Some intra-population diversity in similar contexts, including sex ratios, dietary structure, and lifestyle patterns, may lead to differences in the effect of age structure changes on disease burden. Improved environmental hygiene, the development of vaccines and pathogen-specific tests, and the use of antiviral drugs or antibiotics for treatment are the major factor in reducing the burden of communicable diseases. Despite these successes, the emergence of new diseases, such as the COVID-19 pandemic, increased spread of known pathogens, and increased antibiotic resistance may be a warning that the burden of communicable disease remains a top priority [27]. More specifically, we found that changes in age structure contribute to an increased burden of HIV/AIDS and sexually transmitted infections except in the high-income group. In contrast to the decline in the absolute level of DALYs attributable to age structure in other communicable diseases, the death burden and disability burden of HIV/AIDS and sexually transmitted infections have both increased in the context of population aging. Although antiretroviral therapy (ART) has the major strides in improving the quality of life of people living with HIV, some less developed countries still face the challenge of managing HIV in resource-limited settings [28]. People with HIV who receive ART are living longer and are aging. In Africa, where in the past HIV mainly affected adolescents and adults of reproductive age, the aging HIV epidemic must also be taken into account [29,30]. Care for the elderly infected with HIV and receiving ART is increasingly complex as they suffer from multiple sources of the burden of pain, treatment toxicity, and age-associated comorbidities [31,32]. Therefore, HIV infection has been regarded as a chronic disease [33]. This requires a shift in health care systems and clinical workforce to provide optimal, cost-effective chronic care for elderly people infected with HIV [34]. Although less developed countries are beginning to face the health challenges of population aging, only a few low-income and lower-middle-income countries are aging societies in 2019. In addition to communicable diseases, maternal and neonatal disorders also place a heavy burden on health care systems in low-income and lower-middle-income countries. Maternal and new-born mortality is still the greatest disparity between low-income and high-income countries. Potential reasons may include maternal age, maternal vaccine knowledge, and neonatal vaccination rates [35,36]. Diverse forms of health system interventions and approaches are being implemented in low-income and lower-middle-income countries, mainly including quality improvement, health system strengthening, and implementation of science, towards improved maternal and new-born care. Low-income and lower-middle-income countries should fully consider the characteristics of the three common service delivery-related approaches and the possibilities for synergy between them to improve maternal and new-born outcomes [37].

We found that population aging increases the burden of non-communicable diseases (NCDs), including death and disability from disease, observed globally. From a socioeconomic perspective, the net increase in the burden of NCDs in most low-income countries over the past three decades has not been due to changes in the age structure, but to population growth. It is not hard to understand that the process of population aging is slower in less developed regions since socioeconomic and population aging are often thought to be negatively correlated. Major age-related NCDs in lower-middle-income and upper-middle-income countries include neoplasms, cardiovascular diseases, neurological disorders, diabetes and kidney disease, and musculoskeletal disorders. The difference in high-income countries is that the attributable burden of premature death from neoplasms, and the attributable burden of disability from cardiovascular diseases and neurological disorders, have markedly declined. Even in low-income countries primarily affected by CMNNDs, the burden of mental disorders, substance use disorders, and musculoskeletal disorders attributable to age structure are increasing, consistent with the findings of previous studies [38]. These findings suggest that NCDs once neglected, which were not previously among the major chronic diseases defined by the WHO, now urgently need to be prioritized and allocated resources in healthy aging strategies. Aging is generally to be a major risk factor for NCDs. Although other risk factors associated with NCDs, including excessive smoking and alcohol consumption, poor diet, and physical inactivity, can be effectively addressed for individuals and populations, aging cannot be abolished. The cumulative age-related disease burden consumes high associated costs of diagnosis, treatment, and care, raising serious concerns about the fiscal integrity of health care systems [39]. Although current plans related to non-communicable diseases may not be sufficient to achieve the goal of reducing premature mortality, these could serve as one of the most important steps in achieving healthy aging [40,41]. Cross-cutting strategies that bundle strategies and interventions are crucial. At the broader level, NCDs prevention and control are implemented by identifying health-related social determinants and creating health-promoting environments through people-centred primary health care [41]. For individuals and populations, NCDs-related health interventions, such as eliminating alcohol and tobacco abuse, disease-specific nutrition interventions, and promoting physical activity, need to be started early and often evaluated for their progress and cost-effectiveness [42,43].

Typical discussions on the threat to healthy aging involve conversations about falls and consequential injuries or fractures [44,45]. This is partially supported by our analysis, which found that the increased burden of unintentional injuries can be attributed to age structure and a high proportion of the disability burden. Falls in elderly people are usually a result of joint risk factors, which can be a manifestation of the underlying health disorders specified to this sub-group of the population [46]. The influence of falls on individuals, families, and communities is phenomenal, as it can result in disability and fear, and can trigger a decline in physical function and loss of autonomy [47]. Therefore, falls prevention and management strategies are considered vital for health and aged care systems globally. Aging without injury is possible and there are evidence-based strategies that can reduce the occurrence of falls, lower the risk of injury, and maintain the highest possible level of independence. Broadening awareness of elderly people about falls can empower them to take the actions necessary to reduce their risk of falling [48]. Multifactorial interventions may involve a combination of balance training, medication review, physical activity, and home risk modifications [49,50].

The younger population of the less developed countries might act as a protective factor against age-related disease burden, thus explaining the low burden attributable to the age structure observed in the past 30 years. However, our analysis suggested that the burden of disease due to population aging in low-income and lower-middle-income countries will overtake that of developed countries over the next three decades. The potential reasons for accelerated fertility declines and slower population growth probably are improved female education and the availability of contraception [51]. The World Population Prospects 2019 shows that the proportion of people aged 65 and over will decline in high-income countries in 2050 compared with 2019, while it will nearly triple in low-income and lower-middle-income countries [15]. The double burden of communicable diseases and NCDs is unoptimistic in less developed countries in the context of population aging [52,53]. For both developing and developed countries, the existing age-related disease burden will continue to be prominent in the future [54-57]. The number of DALYs attributable to population aging represents the burden of disease that the health systems must be valued highly worldwide.

This study has the following advantages. First, we quantified the global burden of disease and injury attributable to population aging from 1990 to 2019 which can be compared across regions and periods. Second, the categories of diseases and injuries in this study were without arbitrary restrictions. It is generally accepted that not all causes are age-related, but this view may be biased. In this study not only the changing trends of disease burden were assessed, but also the effects of age, cohort, and period were elucidated by the APC regression model, demonstrating an insight into the analysis of the global health estimates data. Third, this study provides a useful perspective on the future burden of disease due to an aging population globally, which could have implications on health policy and priority setting. The data provided in this study are useful for worldwide authorities to tailor health-related plans for the future.

Several limitations of this study should be acknowledged. First, the COVID-19 pandemic, which outbroke in early 2020, has led to an excessive death burden globally. However, we did not incorporate the excessive mortality of this new infectious disease in the scenario projections. These scenario projections were based on historical trends from 1990 to 2019. Therefore, our projected result may be a conservative estimate of future age-related disease burden. Nevertheless, these scenario projections serve as useful knowledge for identifying key drivers of age-related disease burden and efforts needed to offset the health effects of an aging population.

## CONCLUSIONS

In conclusion, the burden of disease due to population aging is extremely worrying and has a great impact on global health. The direction and intensity of the effects of population aging on the burden of disease vary by region and disease. Making appropriate measures in terms of primary and secondary prevention for specific diseases related to healthy aging is an essential step to addressing the challenges and harnessing the opportunities of population aging.

## Additional material

Online Supplementary Document.
