# Supplementary material for: Measurement and projection of the burden of disease attributable to population aging in 188 countries, 1990-2050: A population-based study
Source: J Glob Health. 2022 Oct 30;12:04093. doi: 10.7189/jogh.12.04093 (PMC9579832; doi:10.7189/jogh.12.04093)
Supplement: Online Supplementary Document. [file jogh-12-04093-s001.pdf]

ONLINE SUPPLEMENTARY DOCUMENT

Title: Measurement and projection of the burden of disease attributable to population aging in 188 countries, 1990-2050: a population-based study

Authors: Jun-Yan Xi[1], Xiao Lin[1], Yuan-Tao Hao[1,2,3,4]

[1]Department of Medical Statistics, School of Public Health, Sun Yat-sen University, Guangzhou 510080, China;

[2]Peking University Center for Public Health and Epidemic Preparedness & Response, Beijing 100191, China;

[3]Sun Yat-sen Global Health Institute, Sun Yat-sen University, Guangzhou 510080, China;

[4]Center for Health Information Research, Sun Yat-sen University, Guangzhou 510080, China.

Contents

Attribution decomposition method of population aging 1

Bayesian age-period-cohort regression model 3

References 4

Attribution decomposition method of population aging

As mentioned in the main paper, we collected annual age-gender-location specific disability-adjusted life years (DALYs) and population estimates from the Global Burden of Disease (GBD 2019). There are a total of 18 age groups (<1, 1–4, 5–9,···, 80–84, 85+ years) involved. We then sorted and organized the data into the following format[1]:

Age group	Year 1（$j=1$）				Year 2（$j=2$）			
	d_{i1}	n_{i1}	r_{i1}	s_{i1}	d_{i2}	n_{i2}	r_{i2}	s_{i2}
1	d_{11}	n_{11}	r_{11}	s_{11}	d_{12}	n_{12}	r_{12}	s_{12}
2	d_{21}	n_{21}	r_{21}	s_{21}	d_{22}	n_{22}	r_{22}	s_{22}
⋮	⋮	⋮	⋮	⋮	⋮	⋮	⋮	⋮
p	d_{p1}	n_{p1}	r_{p1}	s_{p1}	d_{p2}	n_{p2}	r_{p2}	s_{p2}
Total	D_1	N_1	R_1	$S_1 = 1$	D_2	N_2	R_2	$S_2 = 1$

Where, d_{ij}, n_{ij}, r_{ij}, and s_{ij} represent the age-specific number of DALYs, age-specific population size, age-specific DALYs crude rate, and proportion of age-specific population among the total population in the ith age group and the jth year respectively. D_j, N_j, R_j, and S_j represent the totals of the columns.

Then, the main effect of the three factors is represented by M_p, M_s, and M_m respectively, the two-way interaction of the three factors is represented by I_{ps}, I_{pm}, and I_{sm} respectively, and the three-way interaction of the three factors is represented by I_{pSm}, which can be calculated according to the following formula:

$$M_p = \sum_{i=1}^{p} (N_2 - N_1) \times s_{i1} \times r_{i1}$$

$$M_s = \sum_{i=1}^{p} N_1 \times (s_{i2} - s_{i1}) \times r_{i1}$$

$$M_m = \sum_{i=1}^{p} N_1 \times s_{i1} \times (r_{i2} - r_{i1})$$

$$I_{PS} = \sum_{i=1}^{p} (N_2 - N_1) \times (s_{i2} - s_{i1}) \times r_{i1}$$

$$I_{pm} = \sum_{i=1}^{p} (N_2 - N_1) \times s_{i1} \times (r_{i2} - r_{i1})$$

$$I_{sm} = \sum_{i=1}^{p} N_1 \times (s_{i2} - s_{i1}) \times (r_{i2} - r_{i1})$$

$$I_{psm} = \sum_{i=1}^{p} (N_2 - N_1) \times (s_{i2} - s_{i1}) \times (r_{i2} - r_{i1})$$

In the formula, subscripts $p, s,$ and m indicate population size, age structure, and all other causes respectively.

Finally, the contribution of each factor is represented by $AS_{III}, PS_{III}, ASR_{III}$ respectively, and is calculated according to the following formula:

$$AS_{III} = M_s + \frac{1}{2} I_{ps} + \frac{1}{2} I_{sm} + \frac{1}{3} I_{pSm}$$

$$PS_{III} = M_p + \frac{1}{2} I_{ps} + \frac{1}{2} I_{pm} + \frac{1}{3} I_{pSm}$$

$$ASR_{III} = M_m + \frac{1}{2} I_{pm} + \frac{1}{2} I_{sm} + \frac{1}{3} I_{pSm}$$

Bayesian age-period-cohort regression model

The all-cause DALYs was projected using the Bayesian age-period-cohort (BAPC) regression model with integrated nested Laplace approximations (INLA) method[2]. The Bayesian approach attributes separate effects to age, period and cohort, and extrapolates these effects to make projections. Due to the expectation that effects adjacent in time might be similar, smoothing priors are commonly used for age, period, and cohort effect and to project posterior DALYs rates. A standard choice is the second-order random walk (RW2)[3,4]. According to this model, each point of effects is predicted by linear extrapolation from its two immediate predecessors, plus a random variance from a normal distribution with mean zero. The BAPC regression model was developed to predict the number of DALYs by age group in statistical software R, using the BAPC package (R version 4.1.0).

Our primary data source is the GBD 2019. To construct the BAPC model, we introduced the Lexis diagram to the organization of our collected data. The Lexis diagram consists of population estimates and all-cause DALYs by country. Data are given by single calendar year and 17 five-year age groups (0－4, 5－9,⋯, 80－84 years) and an open age group (85+ years). Within the text files, the rows represent periods in increasing order and the columns age groups from young to old. That said, each row represents one period, and each column (besides the first, which shows the year label) represents one age group.

References

1. Cheng X, Tan L, Gao Y, Yang Y, Schwebel DC, Hu G. A new method to attribute differences in total deaths between groups to population size, age structure and age-specific mortality rate. PLoS One. 2019 May 10;14(5):e0216613.

2. Riebler A, Held L. Projecting the future burden of cancer: Bayesian age-period-cohort analysis with integrated nested Laplace approximations. Biom J. 2017 May;59(3):531-549.

3. Shi JF, Cao M, Wang Y, Bai FZ, Lei L, Peng J, Feletto E, Canfell K, Qu C, Chen W. Is it possible to halve the incidence of liver cancer in China by 2050? Int J Cancer. 2021 Mar 1;148(5):1051-1065.

4. Wu X, Du J, Li L, Cao W, Sun S. Bayesian Age-Period-Cohort Prediction of Mortality of Type 2 Diabetic Kidney Disease in China: A Modeling Study. Front Endocrinol (Lausanne). 2021 Oct 29;12:767263.
